# Multi-omics integration reveals gut microbiota dysbiosis and metabolic alterations of cerebrospinal fluid in children with epilepsy

**DOI:** 10.3389/fmicb.2025.1630062

**Published:** 2025-09-11

**Authors:** Feng Li, Dongdong You, Yun Li, Xiaoyu Wang, Zhongdong Lin, Xulai Shi, Zhongshan Li, Jinyu Wu, Zhenwei Liu

**Affiliations:** ^1^Department of Pediatric Neurology, The Second Affiliated Hospital and Yuying Children's Hospital of Wenzhou Medical University, Wenzhou, Zhejiang, China; ^2^Key Laboratory of Laboratory Medicine, Ministry of Education, Institute of Genomic Medicine, Wenzhou Medical University, Wenzhou, Zhejiang, China; ^3^Department of Neonatology, The Second Affiliated Hospital and Yuying Children's Hospital of Wenzhou Medical University, Wenzhou, Zhejiang, China

**Keywords:** epilepsy, gut-brain axis, gut microbiota, cerebrospinal fluid, metabolomics

## Abstract

**Introduction:**

Epilepsy is a complex neurological disorder with an unclear pathogenesis. Emerging evidence suggests that gut microbiota dysbiosis and cerebrospinal fluid (CSF) metabolic alterations play a critical role in epilepsy progression through the gut–brain axis. This study aimed to characterize microbial and metabolic disturbances in pediatric epilepsy and identify potential diagnostic biomarkers through integrative multi-omics analysis of matched fecal and CSF samples.

**Methods:**

In this study, we conducted 16S rRNA gene sequencing on fecal samples from a total of 50 participants including 17 common epilepsy (CEP) patients, 23 refractory epilepsy (REP) patients, and 10 non-epilepsy (NEP) patients, along with untargeted metabolomic analysis on 24 paired CSF samples from REP and NEP groups. Multi-omics integration and a random forest model were applied to assess diagnostic performance, identifying microbial and metabolite signatures associated with epilepsy.

**Results:**

Children with epilepsy (REP and CEP) exhibited distinct gut microbiota dysbiosis. Specifically, multivariable association modeling using MaAsLin 3 identified 13 discriminatory microbial taxa, with *Clostridiales* and *Clostridiaceae* ranking as the most enriched in REP. Functional predictions revealed significant differences in metabolic pathway, alongside disrupted ecological characteristics among epilepsy groups. In addition, CSF metabolomics analysis further revealed key metabolic shifts between REP and NEP, with notable alterations in alpha-Ketoisocaproic acid, alpha-Ketoisovaleric acid, and acetyl-L-carnitine, reflecting distinct metabolic reprogramming in epilepsy. Moreover, correlation analysis revealed strong microbiota-metabolite associations, reinforcing the involvement of the gut-brain axis in epileptogenesis. Independent random forest-based diagnostic models using microbial genera (AUC = 0.913, accuracy = 0.818) or metabolites (AUC = 0.875, accuracy = 0.833) demonstrated high classification accuracy in distinguishing REP from NEP. Notably, the integrated microbiota-metabolite classification model exhibited superior diagnostic performance in REP and NEP groups (AUC = 0.953, accuracy = 0.875), significantly surpassing individual models and highlighting the potential of multi-omics integration for epilepsy diagnostics.

**Conclusion:**

These findings reveal concurrent gut microbiota dysbiosis and CSF metabolic disturbances in epilepsy, underscoring their interrelated roles in epileptogenesis and reinforcing our understanding of microbiome-metabolome crosstalk. The integrated multi-omics model demonstrated superior diagnostic performance, emphasizing its potential for precision biomarker discovery and clinical application in epilepsy stratification and intervention.

## 1 Introduction

Epilepsy represents a complex and heterogeneous neurological disorder characterized by uncontrollable and unpredictable epileptic seizures, with nearly 50 million people affected worldwide and contributing to a significant socioeconomic burden (GBD 2016 Epilepsy Collaborators, 2019). Moreover, approximately one-third of epileptic patients manifests susceptibility to drug resistance after treatment of antiseizure medications (Mesraoua et al., 2023). Despite extensive neurobiological and clinical investigations, the pathogenetic mechanisms underlying epileptogenesis remain incompletely elucidated, reflecting the multifactorial nature of this neurological condition (Scheffer et al., 2017). Given in the clinical complexity and heterogeneity of epilepsy, it is of great significance to unravel the complex etiopathogenes and develop targeted therapeutic strategies, which have always been significant challenge in the field of epilepsy research and therapeutics (Scheffer et al., 2017; GBD 2016 Epilepsy Collaborators, 2019; Mesraoua et al., 2023).

Accumulating evidence have indicated that gut microbiota (GM) could influence brain development and neurological function through the gut-brain axis, which was involved in bidirectional interaction within brain and the gastrointestinal tract (Sorboni et al., 2022; Marizzoni et al., 2023; Rendeli et al., 2023; Nakhal et al., 2024). Several studies of both animal models and human patients showed substantial differences in the GM profiles between epilepsy patients and healthy individuals, as well as modulation in anti-seizure effect of the ketogenic diet (Riva et al., 2025; Peng et al., 2018; Gong et al., 2020; Lee et al., 2020; Gong et al., 2021; Wan et al., 2021; Dong et al., 2022; Zhou et al., 2022). The aforementioned investigations underscore the association of gut bacterial dysbiosis with epilepsy pathogenesis. In addition to gut microbiota, systemic metabolic abnormalities, such as amino acid neurotransmitter metabolism, fatty acid and energy metabolism, have also been consistently found in biofluids or fecal samples of patients with epilepsy (Niu et al., 2022; Wang et al., 2023; Dahlin et al., 2024; Hanin et al., 2024; Lian et al., 2024; Meier et al., 2024). These metabolic profiling analyses also highlighted the perturbed metabolism in the brain of epilepsy sufferers, also indicating the dysregulated metabolites considered as diagnostic and prognostic markers for epilepsy. In fact, gut microbial communities also affect host metabolism with the changes in the gut microbiota often correcting with alterations of metabolites in epilepsy, indicating the role of interplay between the gut microbiome and host metabolism in the pathogenesis of epilepsy (Sorboni et al., 2022; Dahlin et al., 2024; Zou et al., 2024). Therefore, investigation of alteration in both gut microbiota and metabolites would be helpful to comprehensively understand the pathological process of epilepsy.

Recently, the combined analyses of both gut microbiome and metabolome were increasingly rapidly applicated in the exploration of the pathophysiological mechanisms and related diagnostic biomarkers of epilepsy (Zhou et al., 2023; Dahlin et al., 2024; Wan et al., 2024; Zou et al., 2024). Of these multi-omic joint studies, metabolomics analyses have been conducted in serum samples from patients or animal models, but not in cerebrospinal fluid (CSF) from pediatric patients with epilepsy to date. Given its close relationship with the central nervous system (CNS), CSF serves as a crucial and effective source for identifying new potential biomarkers for CNS diseases (Brister et al., 2022). Recent metabolomics analyses of CSF have unveiled considerable differences in metabolite composition between patients with epilepsy and controls, underscoring the link between metabolic imbalances and both physiological and pathological modifications (Akiyama et al., 2020; Niu et al., 2022; Wang et al., 2023; Hanin et al., 2024). In this pilot study, we employed multi-omics analyses to analyze shifts in gut microbiota and CSF metabolites in children with epilepsy in comparison to control groups based on 16S rRNA gene sequencing and ultra-high performance liquid chromatography coupled to mass spectrometry (UHPLC-MS) analysis. The aim was to uncover potential biomarkers for clinical diagnosis and to attempt a comprehensive understanding of the interplay between gut microbiota and CSF metabolites in epilepsy through integrative analysis.

## 2 Materials and methods

### 2.1 Participant recruitment and sample collection

The study participants were recruited from the Neurology Department of the Second Affiliated Hospital and Yuying Children's Hospital of Wenzhou Medical University, from January 2022 to December 2022.

A total of 40 epileptic patients were enrolled and then divided into the 17 common epilepsy (CEP) patients and 23 refractory epilepsy (REP) patients including 16 with CSF collected, according to the diagnosis and classification criteria of epilepsy in ILAE from 2017 (Scheffer et al., 2017). We also enrolled 10 non-epilepsy (NEP) patients matched by age and gender (including 8 with CSF collected), who were ultimately diagnosed with functional headaches (including migraine, cluster headache, emotion-related headache, and sleep-related headache) without positive neurological signs and allocated them to the control group. In addition, lumbar puncture for CSF detection for NEP group was required to rule out both CNS and neurological involvement as determined by the clinicians. The enrollment criteria for the epileptic patients in the study were as follows: (i) age 1–17 years; (ii) stable clinical symptomatology and EEG features from the last 3 months; (iii) recent brain MRI negative for potentially epileptogenic alterations (stroke, tumors, infectious diseases); and (iv) ASMs treatment in both CEP and REP groups given at stable dose from at least 3 months. Exclusion criteria for both the epileptic patients and NEP controls were as follows: (i) clear histories of chronic or allergic diseases; (ii) known inherited metabolic diseases; (iii) treatment with antibiotics, probiotics, or proton pump inhibitors within 3 months before the sample collection; (iv) Definite brain organic lesions caused by sequelae of intracranial infections, mechanical trauma, or spontaneous hemorrhage due to vascular malformations; and (v) treatment with ketogenic diet. According to routine pediatric neurological practice, lumbar puncture is not recommended for children with common epilepsy unless specific clinical concerns arise. Therefore, no CSF samples were collected from CEP patients in this study.

Fecal samples were collected by the parents using a sampling kit (including a sterile collection tube and a cotton swab) and then stored at −80 °C within half an hour. CSF was collected by professional clinicians via lumbar puncture in a sterile polypropylene tube without any additives, labeled, and then stored at −80 °C. This study was approved by the Independent Ethics Committees and Institutional Review Board of the Second Affiliated Hospital and Yuying Children's Hospital, Wenzhou Medical University, and was conducted according to the ethical principle of the Declaration of Helsinki. Prior to enrollment, written informed consent was procured from the patients or their guardians.

### 2.2 Fecal DNA extraction and 16S rRNA gene sequencing

The genomic DNA of Microbial community was extracted from fecal samples using the CTAB/SDS method according to the manufacturer's instructions. DNA concentration and purity were monitored on a NanoDrop 2000 and Qubit 3.0 Spectrophotometer (Thermo Fisher Scientific, Wilmington, United States). The hypervariable V3-V4 region of the 16S rRNA gene was amplified using the specific 341F and 805R primers with the barcode. Sequencing libraries were generated using the NEBNext^®^ Ultra™ II DNA Library Prep Kit (Cat No. E7645), and their quality was evaluated with the Qubit 3.0 Spectrophotometer and the Agilent Bioanalyzer 2100 system. Finally, the libraries were sequenced on an Illumina NovaSeq platform, generating 250 bp paired-end reads.

### 2.3 Gut microbial analysis

The raw sequence data were processed using the QIIME 2 pipeline (Bolyen et al., 2019). With the cutadapt plugin implemented in QIIME2, we removed the adaptor and primer sequences, followed by chimeras, low-quality read ends with a quality score below 35, and identification of amplicon sequence variants (ASVs) using the DADA2 plugin. Taxonomic assignments of ASV representative sequences were conducted based on the SILVA database (version 138) with the RDP Naive Bayesian Classifier algorithm. We removed sequences assigned to mitochondrial and chloroplast for bacteria from the ASV tables for subsequent analysis using the R package microeco (Liu et al., 2021). Subsequent analyses were conducted on taxa with a mean relative abundance of more than 0.01% and present in at least 10% of the samples.

To address variations in sequence depth, we used the rarefy_even_depth method in the “phyloseq” package to rarefy the ASV table to the minimum sequence depth (Mcmurdie and Holmes, 2013). Rarefaction curves were produced for individual samples to evaluate the depth of sequencing by simulating the resampling process based on the microeco package. Three indices of alpha diversity (i.e., Shannon, Pielou_evenness and Richness) were evaluated by diversity functions from the R package vegan. The alpha diversity, community composition and ternary plot were visualized using the “ggplot2” packages in R. Beta diversity matrices were calculated using Bray–Curtis distances, and PCoA plots were generated from Bray–Curtis similarity matrices and visualized using “phyloseq” package. Group significance was determined by analysis of similarities (ANOSIM).

MaAsLin 3 was used to identify microbial features associated with diagnostic groups through multivariable linear modeling (Nickols et al., 2024). Age, sex, and sequencing depth were included as covariates, and associations with both abundance and prevalence were evaluated using False Discovery Rate (FDR) correction (q < 0.05). The gene functions related to the microbial community based on the Kyoto Encyclopedia of Genes and Genomes (KEGG) were predicted by PICRUSt2 (Douglas et al., 2020). Ecologically relevant functional annotation of the gut microbiota was predicted by the trans_func class in the microeco package using the Functional Annotation of Prokaryotic Taxa (FAPROTAX) database (Louca et al., 2016). The prediction of Microbial phenotypic characteristics was analyzed by EasyAmplicon tool based on the BugBase database (Liu et al., 2023).

Random forest models were constructed using differential microbial genera (p < 0.05) identified by Wilcoxon rank-sum tests via the trans_diff function from the microeco package in R, to classify samples according to group labels. Model performance was evaluated using Leave-One-Out Cross-Validation (LOOCV). Probabilistic predictions from the LOOCV procedure were used to compute the Receiver Operating Characteristic (ROC) curve and the corresponding Area Under the Curve (AUC) using the pROC package, in order to evaluate the diagnostic effectiveness of the model. In addition, the Precision-Recall (PR) curve and its corresponding AUC were calculated using the PRROC package to assess performance under class-imbalanced conditions. ROC and PR curves were visualized based on predicted probabilities from LOOCV.

To assess the statistical significance of the model's predictive performance, permutation tests were performed for both AUC and classification accuracy. Specifically, group labels were randomly shuffled 1,000 times, and for each permutation, the random forest model was re-trained using the same feature set and evaluated under the identical LOOCV strategy. The resulting null distributions of AUC and accuracy were then compared to the corresponding values from the original, non-permuted model. Empirical *p*-values were calculated as the proportion of permuted AUC or accuracy values that were greater than or equal to those observed in the original model. For visualization, histograms of AUC and classification accuracy from the 1,000 label-shuffled models were generated using the ggplot2 package, depicting the null distributions. Vertical dashed lines representing the original (non-permuted) AUC and accuracy values were overlaid to highlight their deviation from random expectations.

### 2.4 Metabolite extraction and UHPLC-MS analysis

For CSF metabolomics analysis, 100 μL of CSF samples were placed in EP tubes, resuspended in prechilled 80% methanol with vigorous vortexing, incubated on ice for 5 min, and centrifuged at 15,000 g, 4 °C for 20 min. A portion of the supernatant was diluted to a final concentration of 53% methanol using LC-MS grade water. The samples were then transferred to fresh Eppendorf tubes, centrifuged again at 15,000 g, 4 °C for 20 min, and the supernatant was finally injected into the LC-MS/MS system for analysis. UHPLC-MS/MS analyses were performed using a Vanquish UHPLC system (ThermoFisher, Germany) coupled with an Orbitrap Q Exactive™ HF-X mass spectrometer (Thermo Fisher, Germany).

### 2.5 Data processing and CSF untargeted metabolomics analysis

The raw data files generated by UHPLC-MS/MS were processed using Compound Discoverer 3.3 (CD3.3, ThermoFisher) for peak alignment, peak picking, and quantitation of each metabolite. Main parameters included peak area correction with the first quality control (QC), actual mass tolerance of 5 ppm, signal intensity tolerance of 30%, and minimum intensity. Peak intensities were normalized to total spectral intensity and used to predict molecular formulas based on additive ions, molecular ion peaks, and fragment ions. Peaks were matched with the mzCloud (https://www.mzcloud.org/), mzVault (https://www.mzcloud.org/), and MassList databases for accurate qualitative and relative quantitative results. For non-normally distributed data, relative peak areas were obtained by standardizing according to the formula: sample raw quantitation value/(sum of sample metabolite quantitation value/sum of QC1 sample metabolite quantitation value). Compounds with CVs of relative peak areas in QC samples >30% were removed, and the identification and relative quantification of metabolites were finalized.

Metabolite identification confidence was classified in accordance with the Metabolomics Standards Initiative (MSI). Due to the untargeted nature of the study, most annotated metabolites correspond to MSI Level 2, indicating identification based on high-resolution mass spectra, retention time alignment, and MS/MS spectral matching against reference libraries. MSI Level 1 confirmation, which requires validation using authentic chemical standards, was not feasible for all compounds.

These metabolites were annotated through the KEGG database (https://www.genome.jp/kegg/pathway.html) and the HMDB database (https://hmdb.ca/metabolites). Principal components analysis (PCA) and Partial least squares discriminant analysis (PLS-DA) were conducted using the MetaboAnalyst 6.0 (Pang et al., 2024). In the PLS-DA analysis, metabolites with Variable Importance in Projection (VIP) >1 were considered as important variables for classification. Metabolic pathway analyses associated with these metabolites with VIP > 1 were annotated using MetaboAnalyst 6.0. We applied univariate analysis (*t*-test) to calculate the statistical significance (*p*-value). Metabolites passing the threshold of *p*-values < 0.05 and fold change ≥1.5 or ≤ 2/3 were regarded as statistically different substances. Volcano plots were used to filter metabolites of interest which based on log_2_ (fold change) and -log_10_ (*p*-value) of metabolites by ggplot2 in R. Multivariate ROC analyses on a combination of selected features using random forest analysis were performed to calculate the AUC and analyze the prediction model using MetaboAnalyst 6.0.

### 2.6 Correlational analysis of gut microbiome and CSF metabolome

Spearman correlation analysis was employed to examine the intricate relationship between the microbiome and metabolome. Differential metabolites and key microbiota identified via random forest analysis were selected to compute correlation coefficients and statistical significance using the cal_cor function from the microeco package with visualization through a heatmap. The *p*-value < 0.05 was considered statistically significant for correlation.

## 3 Results

### 3.1 Demographics and clinical characteristics

A total of 40 patients with epilepsy including 17 children with CEP and 23 children with REP, along with 10 NEP controls were enrolled in the study. The NEP cohort included children presenting with functional headache syndromes—such as migraine, emotion-related headache, and sleep-related headache—who exhibited no signs of organic or neurological disease. These participants underwent lumbar puncture as part of standard clinical evaluation to exclude central nervous system involvement. No significant differences were observed among the three groups in terms of age, gender, body mass index, and dietary habits. All clinical data in the cohort are summarized in Table 1 and Supplementary Table S1.

**Table 1:** Demographic and clinical characteristics of the participants in this study.

**Characteristics**	**NEP (*n* = 10)**	**CEP (*n* = 17)**	**REP (*n* = 23)**	***F*/χ^2^**	***p*-value**
Age (year)^a^	11.2 ± 3.58	9.06 ± 3.21	8.52 ± 2.57	2.803	0.071
Gender (males, %)^b^	4 (40)	11 (64.7)	8 (34.8)	3.705	0.186
BMI (kg/m^2^)^a^	17.1 ± 2.73	17.4 ± 2.38	16.2 ± 3.13	0.855	0.432
**Dietray data (** ***n*** **, %)** ^b^					
**Vegetables, and fruit**					
High	4 (40)	4 (23.5)	8 (34.8)	0.844	0.870
Moderate	2 (20)	5 (29.4)	4 (17.4)		
Low	4 (40)	8 (47.1)	11 (47.8)		
**Fried food, and carbonated soft drinks**					
High	5 (50)	8 (47.1)	8 (34.8)	0.776	0.807
Moderate	3 (30)	3 (17.6)	6 (26.1)		
Low	2 (20)	6 (35.3)	9 (39.1)		
**Milk**					
High	4 (40)	4 (23.5)	10 (43.5)	0.774	0.786
Moderate	4 (40)	9 (52.9)	9 (39.1)		
Low	2 (20)	4 (23.5)	4 (17.4)		
**Number of ASMs**					
0		0	0		
1	–	16 (94.1%)	0		
2	–	1 (5.9%)	1 (21.7%)		
3	–	0	15 (65.3%)		
4	–	0	2 (8.7%)		
5	–	0	1 (4.3%)		

NEP, non-epilepsy; CEP, common epilepsy; REP, refractory epilepsy; ASMs, antiseizure medications; BMI, body mass index.

a *p*-value calculated using Analysis of Variance.

b *p*-value calculated using chi-square test.

### 3.2 Gut microbial community composition and diversity

To characterize gut microbiota structure among participants, fifty stool samples were collected from the three groups and analyzed via Illumina MiSeq sequencing of the V3-V4 region of the bacterial 16S rRNA gene. After quality filtering, a total of 3,625,932 high-quality readings were obtained, with an average of 72,519 ± 8,891 sequences per sample (Supplementary Table S2). After denoising and filtering, these sequences were classified into a total of 8,890 ASVs (Supplementary Table S3).

**Figure 1:**
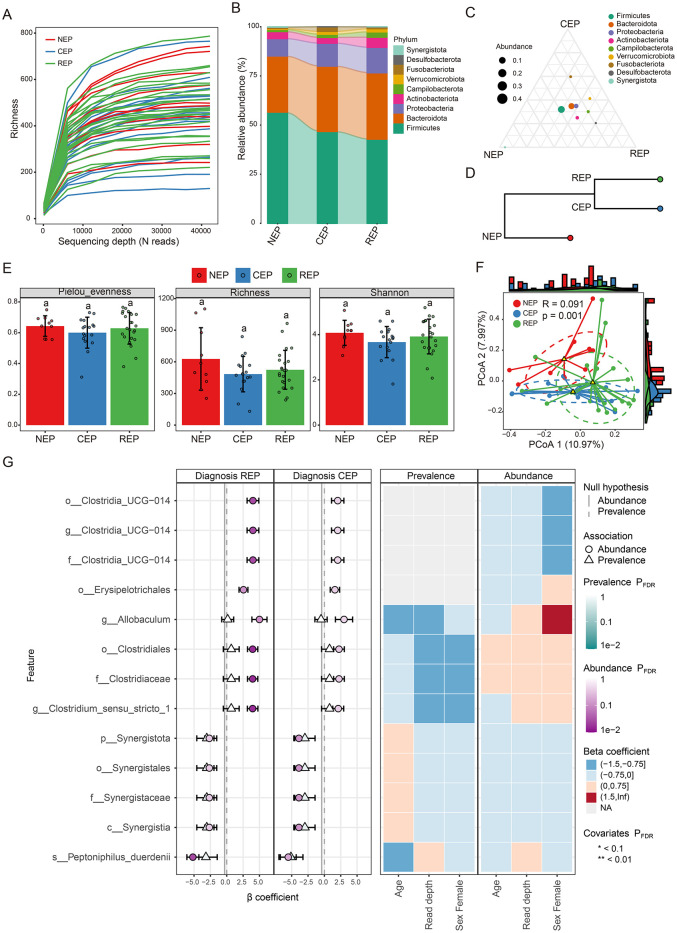
Comparative analysis of gut microbiota composition and diversity across NEP, CEP, and REP groups. **(A)** Rarefaction curve analysis of Richness index for each sample. **(B)** Relative abundances of major bacterial phyla present in the gut microbiota of each group. Bar colors indicate distinct phylum classifications. **(C)** Ternary plot illustrating microbial community composition differences among the three groups. **(D)** Hierarchical clustering of microbial communities based on weighted UniFrac distances **(E)** Comparison of the alpha diversity indices across groups. Statistically significant differences are denoted by different letters, determined via one-way ANOVA. **(F)** Beta diversity analysis using PCoA based on Bray-Curtis distance, indicating significant differences in microbial composition (ANOSIM: *R* = 0.091, *p* = 0.001). **(G)** Visualization of microbial associations with diagnostic groups identified by MaAsLin 3. Circles indicate abundance-based associations, and triangles represent prevalence-based associations. Color intensity corresponds to FDR-adjusted significance levels, with darker shades indicating stronger statistical support. Axes denote effect size distributions for abundance and prevalence models. The right panel displays a heatmap of covariates (e.g., age, read depth, sex), illustrating their adjusted significance across models. Prefixes indicate taxonomic ranks: phylum (p_), class (c_), order (o_), family (f_), genus (g_), and species (s_). NEP, non-epilepsy; CEP, common epilepsy; REP, refractory epilepsy; ANOVA, analysis of variance; PCoA, principal coordinates analysis; ANOSIM, analysis of similarities.

Rarefaction curve analysis of richness, an alpha diversity index, demonstrated sufficient phylogenetic coverage, as curves reached saturation with increasing sequencing depth (Figure 1A). Community composition analysis revealed that bacterial ASVs were affiliated with 54 known phyla, with the top nine phyla represented in a bar graph (Figure 1B; Supplementary Table S4). Of these, five dominant phyla (>1% abundance across all groups) were identified, including *Firmicutes, Bacteroidota, Proteobacteria, Actinobacteriota*, and *Campylobacterota* (Figure 1B). Further examination showed that while *Desulfobacterota, Actinobacteriota*, and *Campylobacterota* were primarily observed in REP, *Fusobacteriota* was predominantly present in CEP (Figures 1B, C), although differences in the average relative abundance of these taxa across groups were not statistically significant (Supplementary Figure S1). Hierarchical clustering analysis revealed that the CEP cohort clustered together with REP, while distinctly separating from NEP (Figure 1D).

Gut microbial diversity analysis showed no significant differences across groups in three alpha diversity indices: Pielou_evenness, Richness, and Shannon index (Figure 1E). However, Principal Coordinates Analysis (PCoA) and ANOSIM analysis based on Bray–Curtis distance indicated significant differences in the gut microbial community composition (*R* = 0.091, *p* = 0.001; Figure 1F). To characterize microbial features associated with diagnostic groups, we applied multivariable association modeling using MaAsLin 3, enabling robust identification of taxa differentially abundant across the REP, CEP, and NEP cohorts while accounting for covariates and data compositionality. The analysis identified 13 taxa significantly associated with diagnostic groups (*q* < 0.05), including eight enriched in REP and five negatively associated with CEP, as detailed in Supplementary Table S5. Notably, members of the Clostridia lineage—including *Clostridiaceae, Clostridiales, Clostridium_sensu_stricto_1*, and *Erysipelotrichales*—exhibited strong positive associations with the REP group. Taxa belonging to the *Synergistia* clade—namely *Synergistia, Synergistaceae, Synergistales*, and *Synergistota*—were negatively associated with CEP. The top 13 group-discriminatory taxa identified by MaAsLin 3 are depicted in Figure 1G, highlighting distinct microbial signatures characteristic of REP and CEP cohorts.

### 3.3 Predicted functional potential of gut microbial community

**Figure 2:**
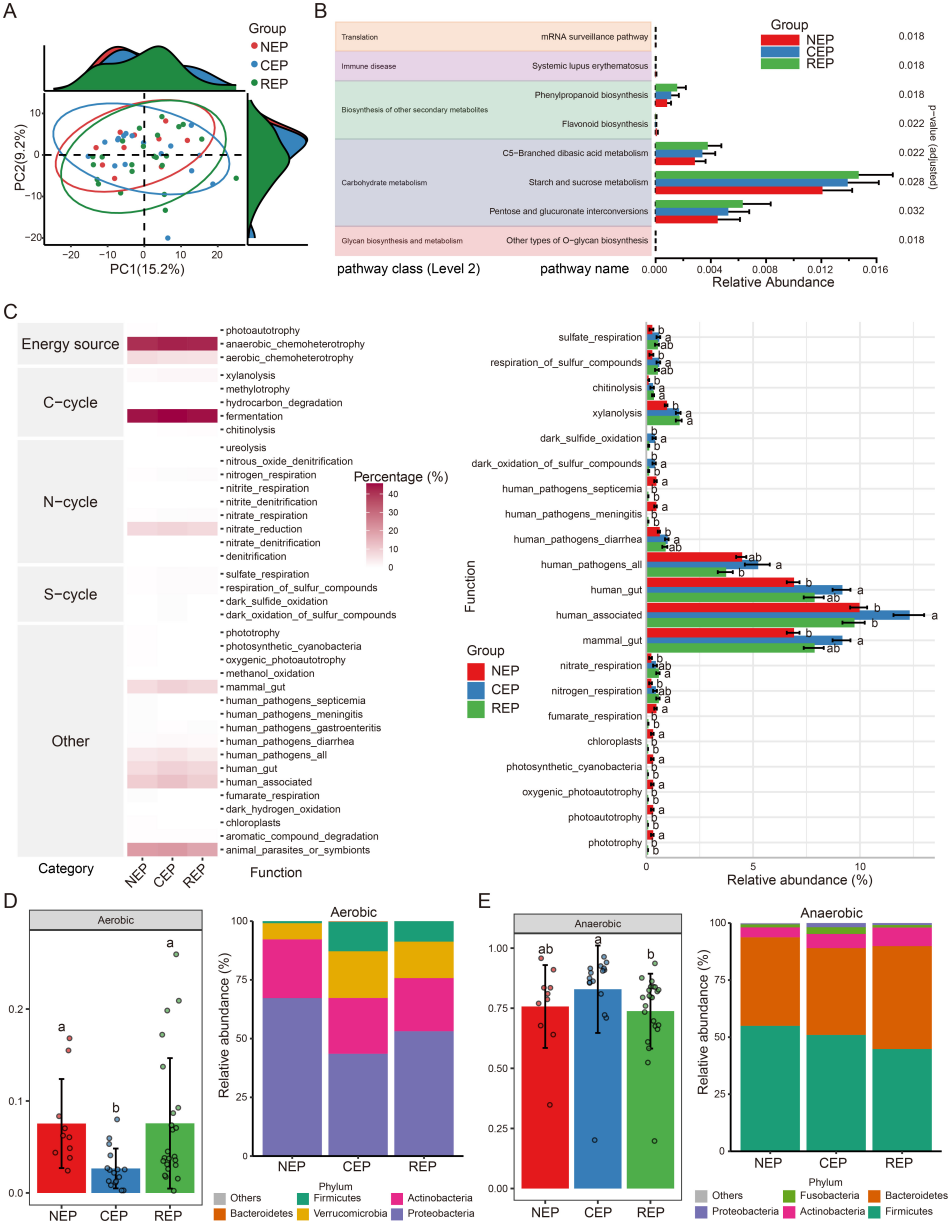
Predicted function analysis of gut microbiota in NEP, CEP and REP. **(A)** PCA analysis of PICRUSt2-predicted functional pathways, showing no significant overall differences among groups. **(B)** Differential functional pathways predicted via PICRUSt2, with significant differences identified using ALDEx2's Kruskal–Wallis test (*p* < 0.05). **(C)** Ecological function analysis using FAPROTAX (left: heatmap of metabolic functions; right: relative abundances of functional groups across groups). ANOVA followed by Duncan's multiple range test determined statistical significance, with different letters indicating significant differences. **(D, E)** Predicted bacterial phenotypes using BugBase. Left panels show phenotype proportions at the phylum level, assessed via Mann-Whitney U test (different letters denote significance). Right panels display the microbial composition of phenotypes at the phylum level. NEP, non- epilepsy; CEP, common epilepsy; REP, refractory epilepsy; PCA, principal component analysis; ANOVA, analysis of variance; FAPROTAX, Functional Annotation of Prokaryotic Taxa; BugBase, bacterial phenotype prediction tool.

PICRUSt2-based functional predictions identified 212 KEGG pathways from filtered ASVs. While PCA analysis indicated no significant differences in the overall composition of predicted functions among the three groups (Figure 2A), ALDEx2's Kruskal–Wallace test revealed statistically significant differences in the relative abundance of eight KEGG pathways spanning five biological processes: translation, immune disease, biosynthesis of other secondary metabolites, carbohydrate metabolism, and glycan biosynthesis and metabolism (Figure 2B).

To further assess potential biotic nutrient cycling mechanisms, we employed the FAPROTAX database, linking microbial taxonomy to functional traits. FAPROTAX analysis identified 38 functional groups associated with carbon (C), nitrogen (N), and sulfur (S) cycling. Among these, fermentation emerged as the most dominant functional category, followed by anaerobic chemoheterotrophy and animal parasites or symbionts (left panel in Figure 2C). ANOVA analysis revealed significant differences in the relative abundance of 21 functional groups (right panel in Figure 2C).

Additionally, bacterial phenotypic characteristics were inferred using the BugBase database, predicting microbial functions across nine phenotypic categories, including pathogenicity, aerobic and anaerobic metabolism, facultative anaerobic capability, mobile elements, biofilm formation, Gram-positive/Gram-negative classification, and oxidative-stress tolerance. BugBase predictions indicated that the REP and NEP groups were enriched in aerobic taxa compared to CEP (Figure 2D). Conversely, the CEP group exhibited significantly higher abundance of anaerobic taxa relative to REP (Figure 2E).

### 3.4 Identification of differential microbial genera and construction of classification models for epilepsy

To explore whether gut microbial composition could be utilized for the diagnosis of epilepsy in children, differential microbial genera were identified using the Wilcoxon rank-sum test via the trans_diff function in the microeco R package. Genera with FDR-adjusted *p*-values < 0.05 were retained as microbial signatures for subsequent classification modeling.

**Figure 3:**
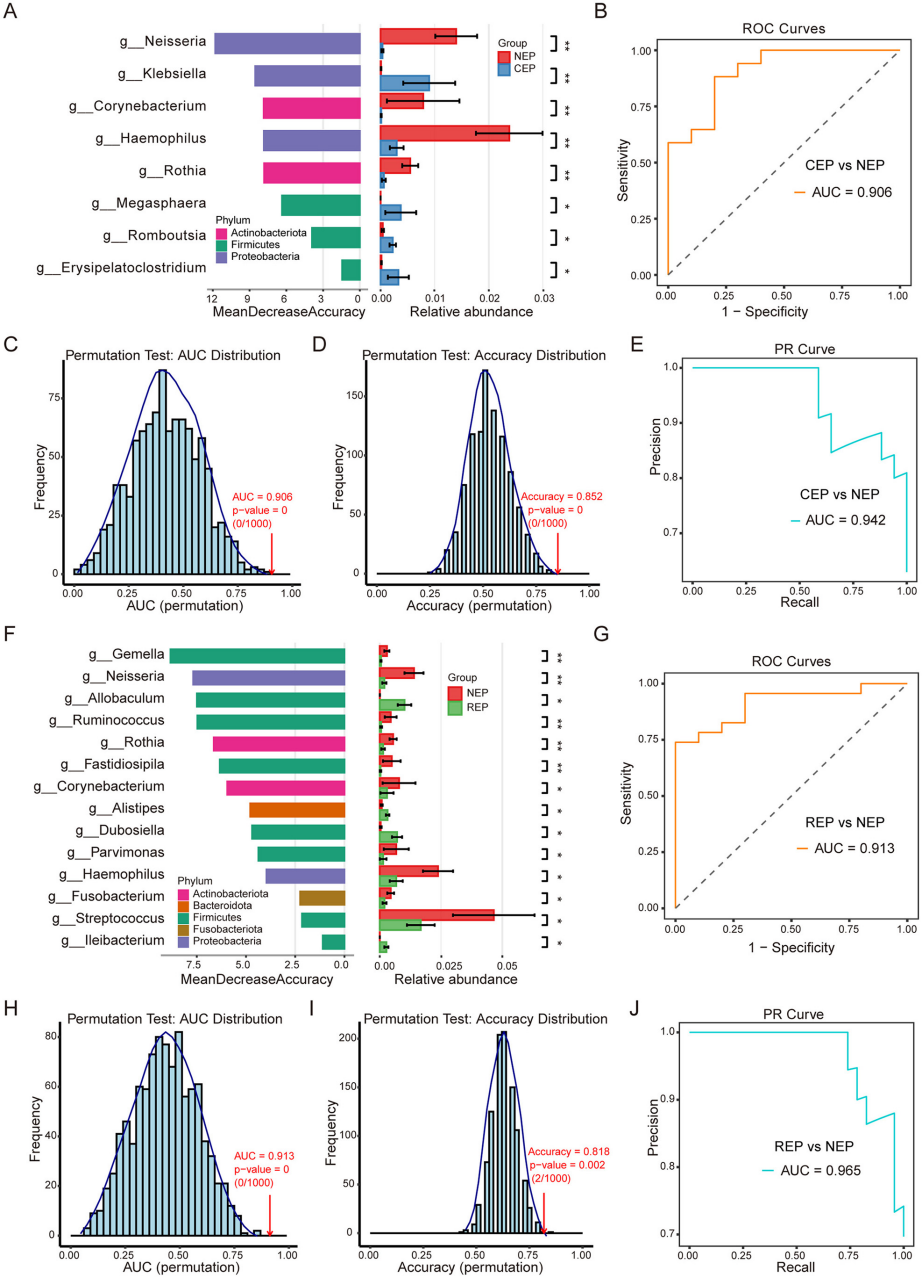
Differential gut microbial genera and random forest classification models for Epilepsy. **(A)** Differential genera between CEP and NEP identified by the Wilcoxon rank-sum test (FDR-adjusted *p* < 0.05). Left: genera ranked by random forest importance (Mean Decrease Accuracy). Right: relative abundance bar plots. Bar colors correspond to the bacterial phylum classification of each genus. **(B)** ROC curve of the CEP vs. NEP classifier (AUC = 0.906). **(C, D)** Permutation tests (*n* = 1,000) for AUC and accuracy, showing distributions under random label shuffling; empirical *p*-values indicate model significance. **(E)** PR curve for the CEP vs. NEP model (AUC = 0.942). **(F)** Differential genera between REP and NEP identified by the Wilcoxon rank-sum test. Left: genera ranked by random forest importance. Right: bar plots showing relative abundance. Bar colors correspond to the bacterial phylum classification of each genus. **(G)** ROC curve for the REP vs. NEP classifier (AUC = 0.913). **(H, I)** Permutation tests (*n* = 1,000) for AUC (*p* = 0) and accuracy (*p* = 0.002) validating model robustness. **(J)** PR curve for the REP vs. NEP model (AUC = 0.965). NEP, non-epilepsy; CEP, common epilepsy; REP, refractory epilepsy; ROC, receiver operating characteristic; PR, Precision–Recall. Asterisks indicate statistical significance: *p < 0.05; **p < 0.01.

There were eight genera with significant differences between CEP and NEP groups (FDR adjusted *p* < 0.05, Wilcoxon rank sum test; Figure 3A). These genera belonged to *Proteobacteria, Firmicutes*, and *Actinobacteriota*, with CEP enriched in genera from *Proteobacteria* and *Firmicutes*, whereas NEP showed a higher abundance of genera from *Proteobacteria* and *Actinobacteriota*. Specifically, *Klebsiella, Megasphaera, Romboutsia*, and *Erysipelatoclostridium* were more prevalent in CEP, while *Neisseria, Corynebacterium, Haemophilus*, and *Rothia* were dominant in NEP (Figure 3A). These differential genera were used as input features for a random forest classification model, which was evaluated through LOOCV. The model demonstrated high discriminatory performance, achieving an AUC of 0.906 for the ROC curve (Figure 3B), an AUC of 0.942 for the PR curve (Figure 3E), and an overall classification accuracy of 0.852. Statistical robustness was confirmed via permutation testing (1,000 iterations), with empirical *p*-values of 0 for both AUC (Figure 3C) and accuracy (Figure 3D), indicating that the observed classification performance was highly unlikely to result from random chance.

Further analysis identified 14 microbial genera displaying significant differences between REP and NEP groups (FDR adjusted *p* < 0.05, Wilcoxon rank sum test; Figure 3F). These genera were distributed across *Firmicutes, Bacteroidota, Proteobacteria, Actinobacteriota*, and *Fusobacteriota*, with REP characterized by an increased presence of *Firmicutes* and *Bacteroidota*, whereas NEP exhibited greater representation of *Firmicutes, Proteobacteria, Actinobacteriota*, and *Fusobacteriota*. Notably, genera such as *Allobaculum, Alistipes, Dubosiella*, and *Ileibacterium* were enriched in the REP group, while the other the 10 genera such as *Gemella, Neisseria*, and *Ruminococcus* were more abundant in the NEP group. A random forest classification model constructed using these genera also demonstrated strong predictive capability, yielding an AUC of 0.913 for the ROC curve (Figure 3G), an AUC of 0.965 for the PR curve (Figure 3J), and an accuracy of 0.818. Permutation tests reinforced model reliability, with empirical *p*-values of 0 for AUC (Figure 3H) and 0.002 for accuracy (Figure 3I), confirming the robustness of the classification approach.

To assess the generalizability of our microbial classification models, we applied the CEP vs. NEP and REP vs. NEP random forest classifiers to an independent pediatric epilepsy 16S rRNA dataset derived from a European cohort (Riva et al., 2025), representing a geographically and ethnically distinct population from our original study. This external cohort includes medication-sensitive (MS) and medication-resistant (MR) patients, as well as healthy controls (HC). The CEP-trained model demonstrated strong discriminatory ability when tested on MS vs HC samples, achieving an AUC of 0.838 and an accuracy of 0.812 (Supplementary Figure S2A). Permutation testing with 1,000 iterations yielded empirical *p*-values of 0 for both metrics, confirming the statistical robustness of the classification (Supplementary Figures S2B, C). Similarly, the REP-trained model achieved an AUC of 0.796 and an accuracy of 0.723 for distinguishing MR from HC samples, with permutation *p*-values of 0 for AUC and 0.004 for accuracy (Supplementary Figures S2D–F), further supporting the reliability of the model's performance in an external cohort. Feature overlap analysis revealed that 7 of the 8 genera used in the CEP classifier (Supplementary Figure S2G) and 9 of the 14 genera used in the REP classifier (Supplementary Figure S2H) were present in the external European dataset. These retained genera were among the top-ranked features in the original models, suggesting that conserved microbial signals contributed to the preserved classification performance.

These findings highlight the potential of genus-level microbial signatures as effective diagnostic markers for epilepsy in children. The high classification accuracy and statistical validation suggest that microbial composition could be leveraged for reliable disease stratification, providing valuable insights into the microbial characteristics distinguishing different clinical conditions.

### 3.5 Untargeted metabolomic alterations in the CSF of children with REP

**Figure 4:**
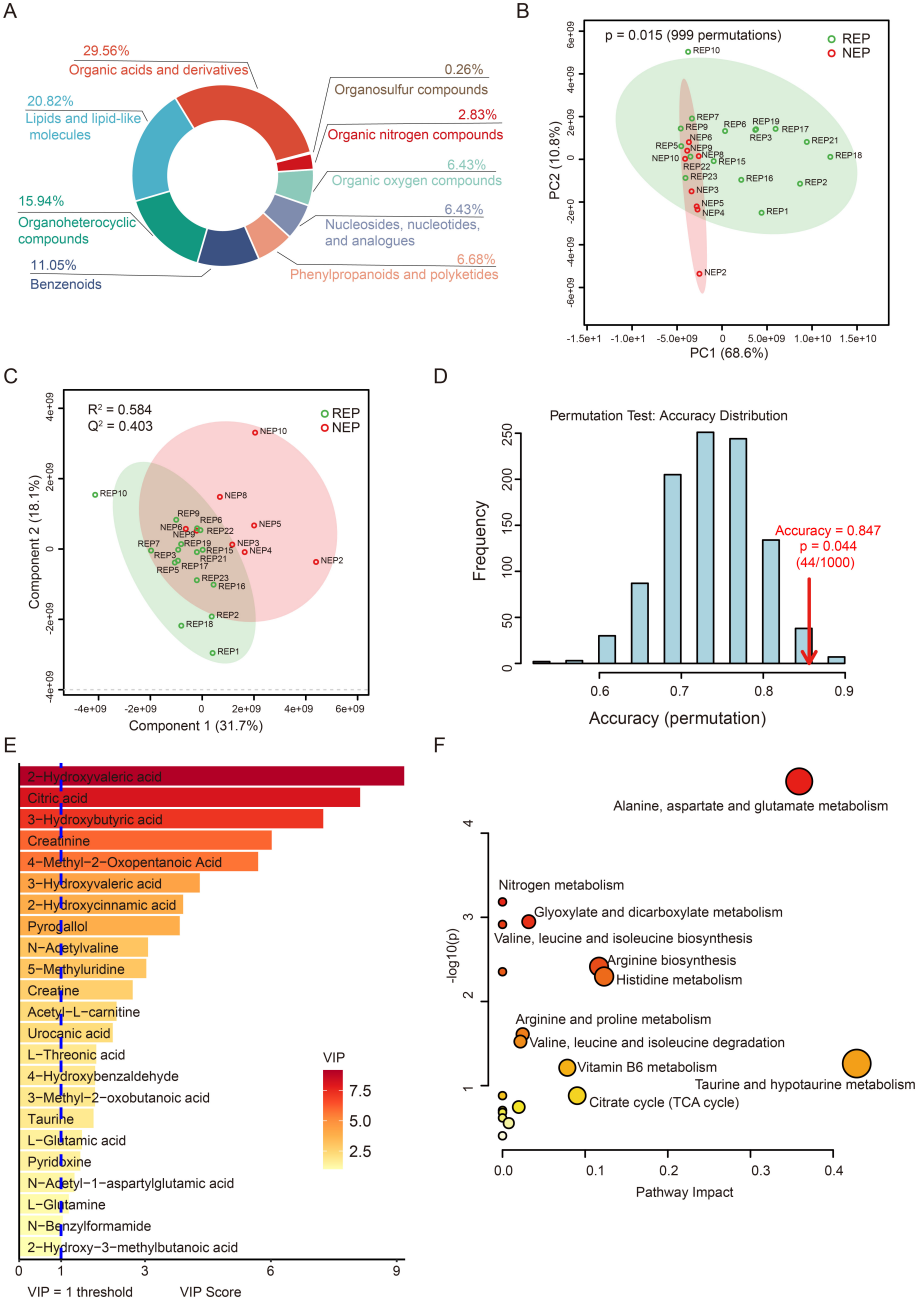
CSF metabolic profile analysis in REP and NEP. **(A)** Donut chart showing HMDB-based classification of all annotated metabolites. **(B)** PCA plot depicting metabolic variation between REP and NEP groups. **(C)** PLS-DA score plot illustrating distinct clustering of REP and NEP samples. **(D)** Validation of the PLS-DA classification model through permutation testing (n = 1000), with an empirical *p*-value of 0.044. **(E)** Important features with VIP > 1 identified by PLS-DA model. **(F)** Pathway analysis of metabolites with VIP > 1 in REP compared to NEP. CSF, cerebrospinal fluid; NEP, non-epilepsy; REP, refractory epilepsy; KEGG, Kyoto Encyclopedia of Genes and Genomes; HMDB, Human Metabolome Database; PCA, principal component analysis; PLS-DA, partial least-squares regression discriminant analysis; VIP, variable importance in projection.

To further explore metabolic dysregulation associated with epilepsy, we conducted untargeted metabolomic profiling of CSF samples from 16 children with REP and 8 children with NEP using LC-MS. Following peak detection, alignment, and deconvolution, a total of 1,082 unique putative metabolites were detected in both positive and negative ionization modes. After filtering exogenous compounds and duplicates, 389 endogenous metabolites were retained based on HMDB annotations (Supplementary Table S6). These metabolites were categorized into 31 secondary categories and further grouped into six primary classifications based on KEGG pathway annotation (Supplementary Figure S3). The majority of metabolites were involved in metabolic pathways, particularly global and overview maps, amino acid metabolism, and carbohydrate metabolism. HMDB-based classification indicated that organic acids and derivatives (29.56%), lipids and lipid-like molecules (20.82%), and organoheterocyclic compounds (15.94%), followed by benzenoids (11.05%), collectively accounted for 77.37% of the annotated metabolites, reflecting a substantial representation of central metabolic intermediates and bioactive compounds (Figure 4A).

Unsupervised principal component analysis (PCA) revealed clear metabolic separation between REP and NEP groups, with the first two principal components explaining 68.6% (PC1) and 10.8% (PC2) of the total variance, respectively. Statistical significance of the observed separation was confirmed via permutation testing (*n* = 999, *p* = 0.015), supporting the existence of distinct metabolic profiles between the two groups (Figure 4B). To further assess class discrimination and identify key metabolites, a PLS-DA model was constructed, demonstrating clear intergroup separation (Figure 4C). Ten-fold cross-validation yielded a classification accuracy of 84.7% (*R*^2^ = 0.584, *Q*^2^ = 0.403; Figure 4C), while ROC analysis returned an AUC of 0.805 (Supplementary Figure S4), indicating robust explanatory and predictive power. Model reliability was further supported by permutation testing (*n* = 1,000), which showed that the observed classification accuracy significantly (0.847) exceeded that of randomly permuted models (*p* = 0.044; Figure 4D).

Based on VIP scores derived from the PLS-DA model, 23 key discriminatory metabolites were identified (VIP > 1.0), including citric acid, taurine, L-glutamic acid, and acetyl-L-carnitine, all of which are involved in energy metabolism and neurotransmitter pathways (Figure 4E). Metabolic pathway analysis using KEGG revealed 19 significantly impacted pathways, with the six most affected (impact score > 0.05) including taurine and hypotaurine metabolism, alanine, aspartate and glutamate metabolism, histidine metabolism, arginine biosynthesis, citrate cycle (TCA cycle), and vitamin B6 metabolism (Figure 4F and Supplementary Table S7). These pathways are functionally linked to neuronal excitability, neurotransmission, and mitochondrial activity.

### 3.6 Metabolite-level classification model

**Figure 5:**
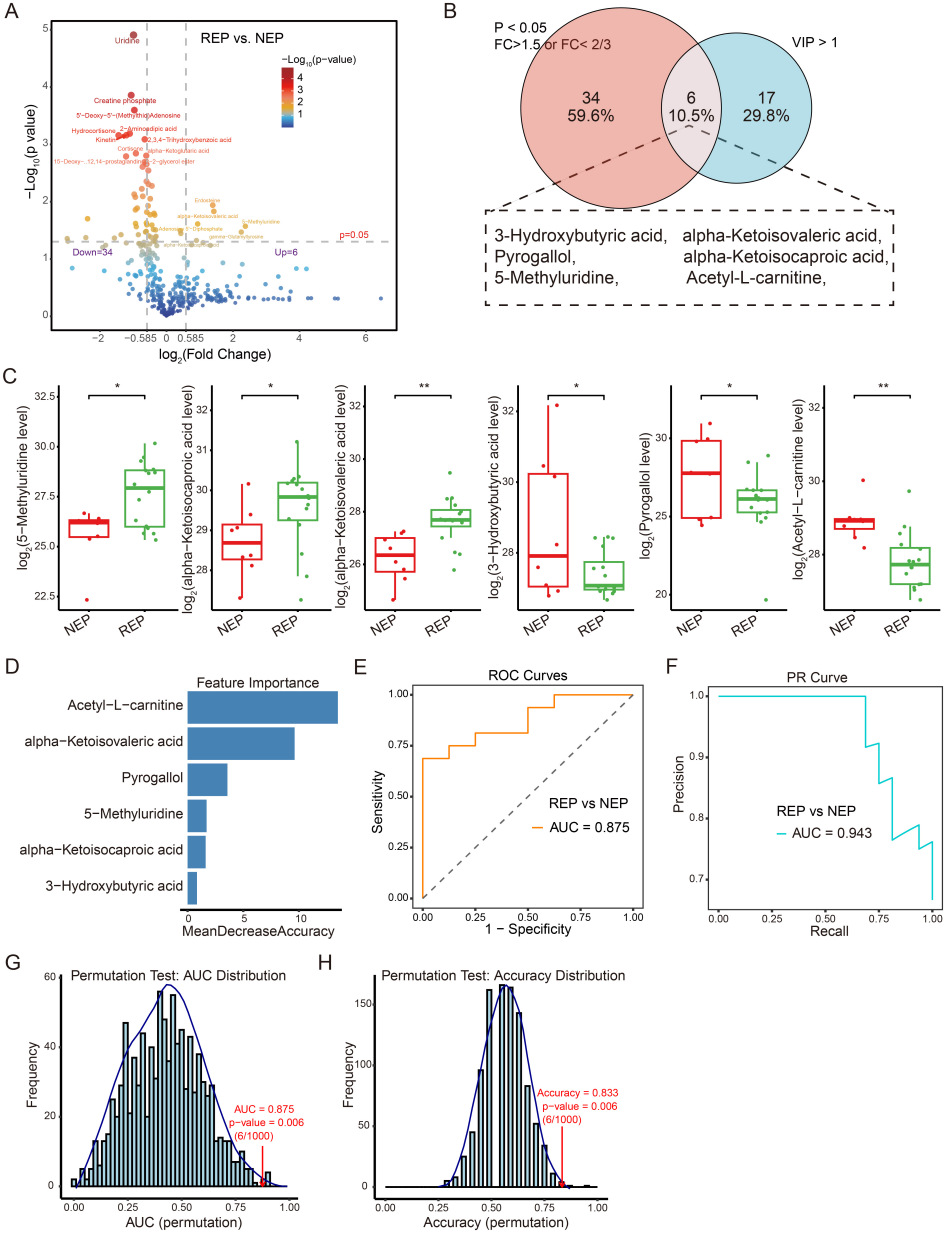
Metabolite-level classification model for REP and NEP. **(A)** Volcano plot showing differential metabolite expression between REP and NEP groups. Red and blue points indicate significantly up- or downregulated metabolites, respectively, based on log2 fold change and -Log10 (*p*-value). **(B)** Venn diagram highlighting the overlap between significantly altered metabolites (*t*-test, *p* < 0.05, fold change > 1.5 or < 2/3) and those with VIP > 1 from PLS-DA. **(C)** Box plots of six key metabolites with statistical significance (*p < 0.05, **p < 0.01) between groups. **(D)** Random forest feature importance ranking of selected metabolites. **(E)** ROC curve (AUC = 0.875) showing model performance in classifying REP vs. NEP. **(F)** PR curve (AUC = 0.943) illustrating precision-recall characteristics. **(G, H)** Permutation test results for AUC and accuracy (1,000 iterations), with empirical *p*-values indicating statistical robustness. CSF, cerebrospinal fluid; NEP, non-epilepsy; REP, refractory epilepsy; PLS-DA, partial least-squares regression discriminant analysis; ROC, receiver operating characteristic; PR, Precision-recall; AUC, area under the ROC curve.

To visualize metabolite expression differences between children with REP and NEP controls, an independent *t*-test was conducted. This univariate analysis identified 40 significantly altered metabolites (*p* < 0.05), including six upregulated metabolites (fold change > 1.5) and 34 downregulated metabolites (fold change < 2/3) in the REP group (Figure 5A). To further refine feature selection for diagnostic modeling, significantly altered metabolites from the *t*-test were intersected with those exhibiting VIP scores > 1 in the PLS-DA mode. This approach yielded six shared metabolites for model construction (Figure 5B), comprising three elevated and three reduced metabolites in REP compared to NEP (Figure 5C).

These six discriminatory metabolites were used to construct a classification model employing a random forest algorithm with LOOCV to evaluate their predictive capacity (Figure 5D). The model exhibited promising diagnostic potential, achieving an AUC of 0.875 for ROC curve (Figure 5E), an AUC of 0.943 for PR curve (Figure 5F), and an overall classification accuracy of 0.833. Model reliability was further assessed through permutation testing (*n* = 1,000), where sample labels were randomly shuffled while preserving model architecture. The original model consistently outperformed the permuted models, yielding empirical *p*-values of 0.006 for both AUC and accuracy (Figures 5G, H), supporting reliability and non-random predictive ability of the model. These findings highlight the diagnostic relevance of metabolite-based classification.

### 3.7 Correlation and classification analysis of gut microbiota and metabolites

**Figure 6:**
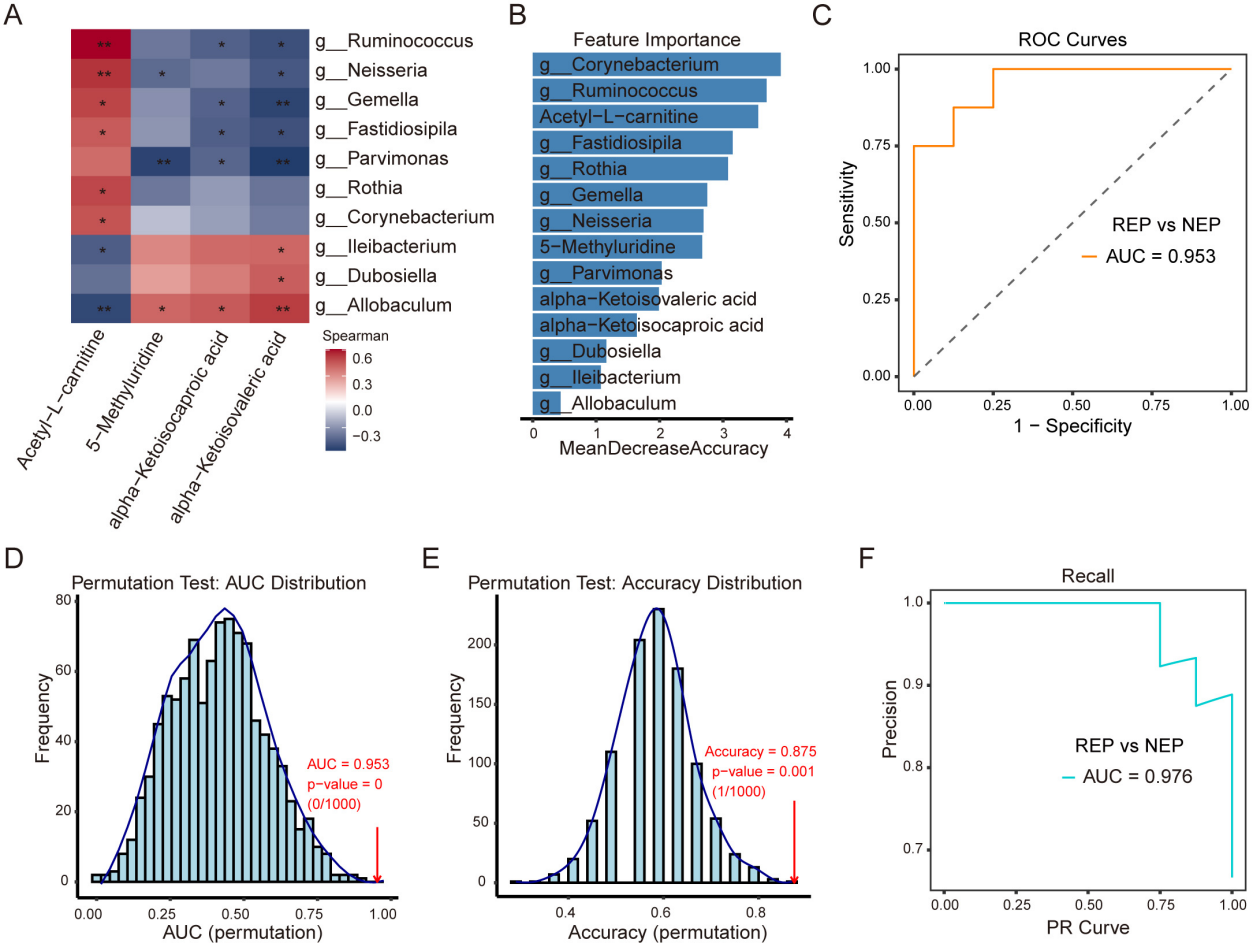
Correlation analysis of CSF metabolites with gut microbiota and classification model performance. **(A)** Spearman correlation heatmap between four CSF metabolites and 10 gut bacterial genera. Red and blue shading indicate positive and negative correlations, respectively, with asterisks marking statistical significance (*p < 0.05; **p < 0.01). **(B)** Feature importance plot ranking bacterial genera and metabolites based on mean decrease in accuracy from the random forest model. **(C)** ROC curve (AUC = 0.953) illustrating model performance in distinguishing REP from NEP. **(D, E)** Permutation tests for AUC and accuracy (1,000 iterations), with empirical *p*-values indicating statistical robustness. **(F)** PR curve (AUC = 0.976) showing precision-recall characteristics. CSF, cerebrospinal fluid; NEP, non-epilepsy; REP, refractory epilepsy; ROC, receiver operating characteristic; PR, Precision-recall; AUC, area under the curve.

To investigate potential associations between metabolite alterations and gut microbiota composition, Spearman's rank correlation analysis was performed. This analysis examined correlations between six shared metabolites and the relative abundance of 14 differential bacterial genera (identified by the Wilcoxon rank-sum test) in paired fecal and CSF samples from 16 children with REP and 8 with NEP. Applying a selection criterion requiring at least one significant correlation (p < 0.05) for both metabolites and bacterial genera, four metabolites exhibited significant associations with 10 bacterial genera (Figure 6A). Correlation analysis showed that 5-Methyluridine, alpha-Ketoisocaproic acid (KIC), and alpha-Ketoisovaleric acid (KIV) had strong positive correlations with three bacterial genera while exhibiting negative associations with seven others, though some of these negative correlations were not statistically significant (Figure 6A). Notably, these three metabolites were more abundant in the REP group compared to NEP (Figure 5C). Conversely, Acetyl-L-carnitine exhibited negative associations with three bacterial genera and positive correlations with seven, although two of these positive correlations were not statistically significant, and its abundance was significantly higher in NEP (Figures 5C, 6A).

To assess the combined discriminatory power of these microbial and metabolic features, a random forest model was constructed using the 10ten bacterial genera and four significantly correlated metabolites, employing LOOCV for evaluation. Feature importance rankings (Figure 6B) identified *Corynebacterium, Ruminococcus*, and Acetyl-L-carnitine as the top three contributors to classification performance. The model demonstrated strong discriminatory capability, as evidenced by an ROC curve with an AUC of 0.953 (Figure 6C). Model robustness was further evaluated via permutation testing (1,000 iterations). The AUC permutation test yielded a *p*-value of 0 (0/1,000), indicating that the observed AUC was highly unlikely to occur by chance (Figure 6D). Similarly, accuracy distribution analysis showed the original model achieving an accuracy of 0.875 (Figure 6E), significantly outperforming permuted models (*p* = 0.001, 1/1,000). The PR analysis further reinforced classification performance, with an AUC of 0.976 (Figure 6F), reflecting high precision and recall in distinguishing REP from NEP. These findings highlight the strong interplay between gut microbiota and CSF metabolites, suggesting that their combined signatures may serve as effective biomarkers for distinguishing REP from NEP. The robustness of the classification model further supports the potential utility of microbial-metabolite interactions in disease stratification.

## 4 Discussion

Growing evidence suggests that gut microbial dysbiosis and metabolic impairments contribute to epilepsy pathogenesis. While previous studies have primarily relied on single-omics approaches, limiting insights into microbiota-metabolite interactions, this study employed a multi-omics framework integrating gut microbiota profiling and untargeted CSF metabolomics in pediatric epilepsy. We observed significant dysregulations in microbial composition and metabolic pathways, with beta diversity analysis revealing distinct microbial structural shifts. Multivariable association modeling using MaAsLin 3, combined with random forest classification, identified key microbial genera that distinguish REP and CEP patients from NEP controls, demonstrating promising diagnostic potential. Functional predictions using PICRUSt2 and FAPROTAX highlighted disruptions in carbohydrate metabolism and immune-related pathways, reinforcing the role of microbiome alterations in epilepsy pathophysiology. Similarly, CSF metabolomics analysis uncovered key metabolic disturbances, particularly in neurotransmitter metabolism and energy pathways, supporting their link to neuronal hyperexcitability. A metabolite-based diagnostic model constructed using six discriminatory metabolites exhibited high predictive accuracy (AUC = 0.875, accuracy = 0.833). Most notably, integrating microbial and metabolite features into a multi-omics classification model significantly enhanced diagnostic performance (AUC = 0.953, accuracy = 0.875), underscoring the exceptional discriminatory capability of microbiota-metabolite signatures. These findings underscore the strong interplay between gut microbiota and CSF metabolites, highlighting their potential as effective biomarkers for disease stratification and precision diagnostics in epilepsy.

The associations between gut microbial alpha diversity and refractory epilepsy or drug-sensitive epilepsy have been controversial because of inconsistent results reported by several previous studies (Peng et al., 2018; Gong et al., 2020; Lee et al., 2020; Gong et al., 2021; Wan et al., 2021; Zhou et al., 2022). However, relatively reduced alpha diversity might represent the characteristics of bacterial community associated with epilepsy reported by a previous study through control over the confounding factors, such as age and recent antiseizure medication exposure (Lee et al., 2020). Although three indexes of alpha diversity did not reveal statistically significant changes in the present study, the slightly decreased tendency seemed to occur in two epilepsy groups compared to control. Furthermore, we observed significant differences in beta diversity among three groups, reflecting a noticeable intra-individual alteration of microbial structure and the state of gut dysbiosis, which had been previously reported by a considerable number of studies (Peng et al., 2018; Gong et al., 2020; Lee et al., 2020; Gong et al., 2021; Zhou et al., 2022).

Our results showed *Fusobacteria* were abundant in the CEP, which was also reported in a previous study. The Fusobacteria was also found to mostly exist in inflamed gut mucosa, thus considered to be pathogenic to human. Apart from the observed differences in the relative abundance of *Fusobacteria*, two taxa—including *Clostridiaceae* and *Clostridiales*—were significantly increased in the REP group and identified as top-ranking features by multivariable association modeling using MaAsLin 3. This enrichment of *Clostridiales* and *Clostridiaceae* aligns with broader compositional shifts previously reported in seizure-prone WAG/Rij rats at 4months of age, indicating a potential association between these taxa and disease susceptibility (Citraro et al., 2021). Additionally, *Erysipelotrichales* have been implicated in refractory epilepsy in children and adult-onset epilepsy, further supporting the relevance of microbial alterations in seizure pathophysiology (Peng et al., 2018; Dong et al., 2022). Collectively, these findings suggest that specific gut bacterial lineages may contribute to seizure susceptibility.

Functional prediction analysis by PICRUSt2 in our study revealed that three 3rd-level KEGG pathways of carbohydrate metabolism were significantly active in two epilepsy group, which was also found in the previous studies on epilepsy and gut microbiota (Gong et al., 2020; Lee et al., 2020; Dong et al., 2022). The disturbance of carbohydrate metabolites was considered to be closely related to the onset of epilepsy. Interestingly, flavonoid biosynthesis revealed a significantly enrichment in control group. By inhibiting the synthesis and release of key inflammatory mediators and inflammatory signaling pathways such as NF-κB, MAPK, JNK, and JAK, flavonoids can pass through the blood–brain barrier and offer neuronal protection, which support the potential of flavonoids in epilepsy treatment (Kiriyama et al., 2024; Zhang et al., 2024).

As the main component of the CNS extracellular space, CSF may contain several informative indicators or biomarkers in neurological disease diagnosis and pathogenesis exploration (Shahim et al., 2013). Several recent studies reveal that the abnormal expression of metabolites in CSF might be associated with epilepsy in children or adults (Akiyama et al., 2020; Niu et al., 2022; Wang et al., 2023; Hanin et al., 2024). In parallel with the alterations noted in the GM of refractory epilepsy, we performed a comparative investigation of metabolic abnormalities in CSF between the REP and NEP cohorts. In line with previous studies, we also found several altered metabolites and pathways which might be involved in the pathogenesis and prognosis of refractory epilepsy or neurological disorders, such as alanine, aspartate and glutamate metabolism (Guo et al., 2023; Wang et al., 2023; Hanin et al., 2024; Lian et al., 2024; Meier et al., 2024); arginine biosynthesis (Wang et al., 2023; Meier et al., 2024); vitamin B6 metabolism (Mastrangelo and Cesario, 2019; Stevelink et al., 2019; Chi et al., 2022); and citrate cycle (TCA cycle) (Guo et al., 2023; Wang et al., 2023).

Alanine, aspartate, and glutamate metabolism has been explored in the context of epilepsy, with some studies focusing on glutamate metabolism, in which alterations in glutamate levels have been observed frequently in patients with epilepsy (Guo et al., 2023; Wang et al., 2023; Hanin et al., 2024; Lian et al., 2024; Meier et al., 2024). Several metabolite alterations occur in the alanine, aspartate, and glutamate metabolism of the patient with refractory epilepsy, despite the fact that our investigation did not find any significant differences in glutamate levels between the two groups. These metabolites with VIP >1 in the PLS-DA model include lower levels of N-acetyl-1-aspartylglutamic acid (NAAG), L-glutamine and citric acid, as well as increased levels of L-glutamic acid. NAAG, the predominant dipeptide in the brain, serves a neuromodulatory function in glutamatergic synapses and has been implicated in several neurological and psychiatric disorders, including epilepsy, schizophrenia, and stroke (Morland and Nordengen, 2022). Reduced glutamine levels, potentially linked to a glutamine synthetase deficit, impair the glutamate–glutamine cycle, as observed in patients with status epilepticus (Hanin et al., 2024) and in a rat model of epilepsy (Swamy et al., 2011). Lower concentrations of citric acid, an essential intermediate in the TCA cycle, have been associated with resistance to drug treatment in epilepsy (Guo et al., 2023). Similarly, dysregulation of citric acid cycle intermediates was found to alter TCA cycle metabolism in the SLC13A5 deficient patients with epilepsy (Bainbridge et al., 2017). In addition, pyridoxine (vitamin B6) is crucial for the synthesis of neurotransmitters gamma-aminobutyric acid (GABA) and monoamines (Chi et al., 2022). Disruptions in vitamin B6 metabolism can result in neuronal migration defects and dysplasia, which are observed in individuals with these metabolic perturbations (Stevelink et al., 2019; Wilson et al., 2019; Chi et al., 2022). In addition, alterations in the arginine biosynthesis pathway have been observed in the CSF of children with poor outcomes following status epilepticus, suggesting its involvement in the pathogenesis of a poor prognosis in status epilepticus (Wang et al., 2023). These metabolic abnormalities and perturbed metabolic pathway may contribute to early onset epileptic encephalopathy.

Through combination of PLS-DA model and *t*-test, we identified six key metabolites which exhibited a clear discrimination between the patients with REP and the NEP controls. Correlation analysis between these differential metabolites and distinct microbial genus features showed three elevated differential metabolites correlated positively with three bacterial genera. KIC, one of branched-chain keto acids, is recognized as a significant neurotoxic metabolite due to its elevated plasma levels being linked to the onset of neurological symptoms (Farias et al., 2021). Intracerebroventricular administration of KIC in a rat model demonstrated that KIC induces oxidative damage, impaired habituation memory, and long-term memory deficits, which may be associated with neurodegenerative and neuropsychiatric disorders, as well as cognitive impairment (Taschetto et al., 2017). Another *in vivo* study in rats showed that 6 h post-injection, KIC significantly cause lipid oxidative damage and impair antioxidant defenses, suggesting that KIC may disrupted redox homeostasis, associated with neural damage (Zemniacak et al., 2024). In addition to KIC, our analysis identified KIV, another branched-chain keto acid, as a putative metabolite with elevated levels. KIV was found to induce dose-dependent seizure-like behavior in rats, suggesting that elevated KIV concentrations in the brain may trigger seizures or severe neurological symptoms in patients (Coitinho et al., 2001; Amaral and Wajner, 2022). Similarly, this metabolite was also found increased in the CSF of patients with mild cognitive impairment and Alzheimer's disease (Berezhnoy et al., 2023). Therefore, the increased KIC and KIV levels in the REP might enter the brain through the blood-brain barrier led to the deleterious effects in the neural cells. Among bacterial genera positively associated with these elevated CSF metabolite changes, the genera *Allobaculum* had been shown to potentially pathogenic effects and infection risks with relation to neurological disorder (Liao et al., 2024), inflammation response (Rice et al., 2022) or oxidative stress (Liao et al., 2024). These evidences suggest that both the perturbed functional metabolic patterns and dysregulation of homeostasis of gut microbial communities may exert a common influence on brain development, mood, and behavior.

Apart from the elevated metabolites correlating with bacterial genera, we also observed reduced molecule linked with bacterial genera. Acetyl-L-carnitine (ALCAR), an endogenous transport molecule, was considered as antioxidants, neuromodulators and neuroprotectors with essential roles in protection of developing brain (Ferreira and Mckenna, 2017). A recent study on mice model with temporal lobe epilepsy induced by kainite suggest ALCAR administration could properly attenuate intensity of seizures and also incidence of kainate-induced status epilepticus (Tashakori-Miyanroudi et al., 2022). The aforementioned evidence underscores the neuroprotective effect and potential therapeutic role of the Acetyl-L-carnitine in epilepsy. In fact, the lower levels of ALCAR in epilepsy patients may result in higher brain ammonia levels, which can lead to seizures due to increased glutamate production and activation of N-methyl-D-aspartate receptors (Maldonado et al., 2020). Additionally, we have identified several gut microbial genera positively associated with the beneficial CSF metabolite change. These gut microbiotas were characterized by common health-associated commensals from the genera such as *Rothia, Neisseria*, and *Ruminococcus*, all of which showed strong correlation with the beneficial CSF metabolite (ALCAR), although several species of these genera might be associated with several diseases. Several other bacteria were yet less well-characterized for human health such as *Gemella* and *Fastidiosipila*.

It is important to acknowledge that the overall sample size in this study was relatively limited, particularly for paired fecal and CSF analyses. This constraint stems from the ethical and logistical challenges of obtaining lumbar puncture specimens from pediatric patients. Nonetheless, strict inclusion and exclusion criteria—along with careful age and gender matching—were applied to minimize biological and clinical heterogeneity. Although formal power analysis was not conducted due to practical constraints, the observed effect sizes and model performance metrics suggest biological relevance warranting follow-up validation. Despite the modest cohort size, our multi-omics analysis revealed robust and statistically significant microbial and metabolic distinctions among groups. Notably, machine learning–based classification models demonstrated high accuracy in disease stratification, underscoring the biological relevance of the identified features. To mitigate potential overfitting due to the limited sample-to-feature ratio, we employed leave-one-out cross-validation, permutation testing, and statistical feature selection. However, we acknowledge that the risk of overfitting cannot be entirely excluded, and future validation in independent cohorts will be essential to confirm generalizability. We consider this work an exploratory investigation and recognize that validation in larger, multicenter cohorts will be essential to support generalizability and clinical translation.

In addition, we acknowledge that metabolite identification in our untargeted LC-MS/MS analysis was based on spectral matching and retention time alignment, corresponding to Metabolomics Standards Initiative (MSI) Level 2 confidence. While confirmation using authentic standards (MSI Level 1) was not feasible in this pilot study, rigorous quality control—including fragment ion validation and filtering of high-variability features—was implemented to strengthen reliability. Key metabolites such as KIC, KIV, and acetyl-L-carnitine were consistently identified across statistical workflows and are supported by prior literature as relevant to epilepsy pathophysiology, reinforcing their biological plausibility. Targeted metabolomics validation and quantitative assessment will be prioritized in follow-up studies to confirm identity and further evaluate diagnostic utility.

While the current study is observational and does not establish causality, several identified metabolites have previously demonstrated seizure-inducing effects in animal models, particularly KIC and KIV. These findings suggest potential mechanistic relevance in epileptogenesis. Building on this, we are initiating experimental validation using preclinical epilepsy models to further investigate their causal roles in neuronal excitability and disease progression. These findings remain associative and serve as hypothesis-generating observations to inform future mechanistic studies. Likewise, we observed strong associations between specific microbial genera, including *Allobaculum* and *Ruminococcus*, and CSF metabolite profiles. To explore their functional relevance, these taxa will be introduced into mouse models of epilepsy under gnotobiotic or antibiotic-depleted conditions. Moreover, fecal microbiota transplantation, followed by CSF metabolomic profiling, represents a compelling strategy to evaluate whether microbial shifts can directly influence CNS metabolic phenotypes. We plan to explore this approach in future experiments as a means to delineate causal links within the gut–brain axis and to assess microbial contributions to epileptogenesis.

**Figure 7:**
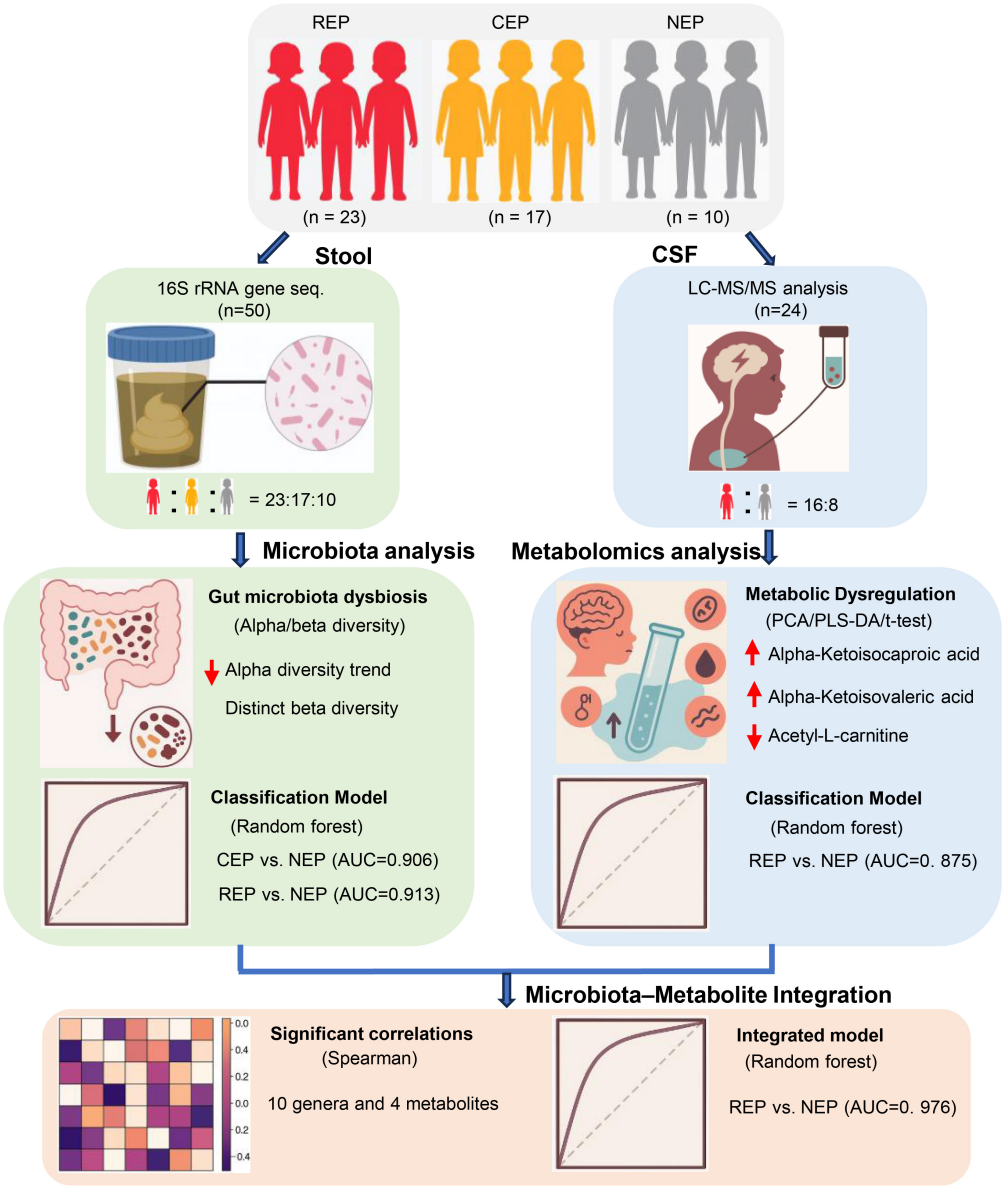
Schematic overview of study design, analytical workflow, and key findings. This figure summarizes the participant groups (REP, CEP, and NEP), sample collection procedures (fecal and CSF), key analytical methods (16S rRNA sequencing and CSF metabolomics), and major integrative steps, including diagnostic modeling and microbiota–metabolite correlation analysis. Classification performance and cross-omics findings are visually integrated to support interpretation of the multi-omics framework. REP, refractory epilepsy; CEP, common epilepsy; NEP, non-epilepsy; CSF, cerebrospinal fluid.

Additional limitations include the composition of the control group in the CSF metabolomics cohort. Due to the ethical constraints of performing lumbar puncture in healthy pediatric populations, our NEP group consisted of children with functional headache syndromes (e.g., migraine, emotion-related headache, sleep-related headache) but without neurological or structural abnormalities. While these individuals were neurologically asymptomatic aside from headache and underwent CSF testing to exclude CNS pathology, their symptomatic status may introduce confounding factors. In future studies, recruiting asymptomatic controls where feasible will help improve interpretability of metabolomic comparisons. Furthermore, the lack of metabolomic data from CEP cases limited direct comparisons across epilepsy subtypes. As CSF sampling is not routinely performed in CEP patients for clinical reasons, this gap reflects a real-world constraint that should be addressed in subsequent investigations. These factors may necessitate cautious interpretation of CSF findings. Finally, while 16S rRNA sequencing and untargeted metabolomics offer broad characterization, future integration of shotgun metagenomics and targeted metabolic profiling will be critical for refining mechanistic insights and improving biomarker resolution. A schematic overview of the study design, analytical workflow, and key findings is presented in Figure 7 to aid interpretation of the integrative multi-omics approach.

## 5 Conclusion

Our study reveals significant gut microbiota dysbiosis and altered CSF metabolite profiles in pediatric epilepsy, particularly in REP. Key microbial taxa and metabolites exhibited promising diagnostic potential, with multi-omics integration uncovering robust microbiota-metabolome interactions. Most notably, the combined microbiota-metabolite classification model demonstrated exceptional accuracy, reinforcing its potential as a powerful biomarker-driven approach for epilepsy diagnosis. These findings provide valuable insights into gut-brain axis disruptions and highlight promising avenues for precision diagnostics and targeted therapeutic strategies in epilepsy management.

## Data Availability

The datasets presented in this study can be found in online repositories. The data is available here: https://www.ncbi.nlm.nih.gov/sra/PRJNA1273592.
