# Supplementary material for: Multi-omics integration reveals gut microbiota dysbiosis and metabolic alterations of cerebrospinal fluid in children with epilepsy
Source: Front Microbiol. 2025 Sep 11;16:1630062. doi: 10.3389/fmicb.2025.1630062 (PMC12461256; doi:10.3389/fmicb.2025.1630062)
Supplement: Supplementary file 8 [file Data_Sheet_1.docx]

Supplementary Material

# Supplementary Figures


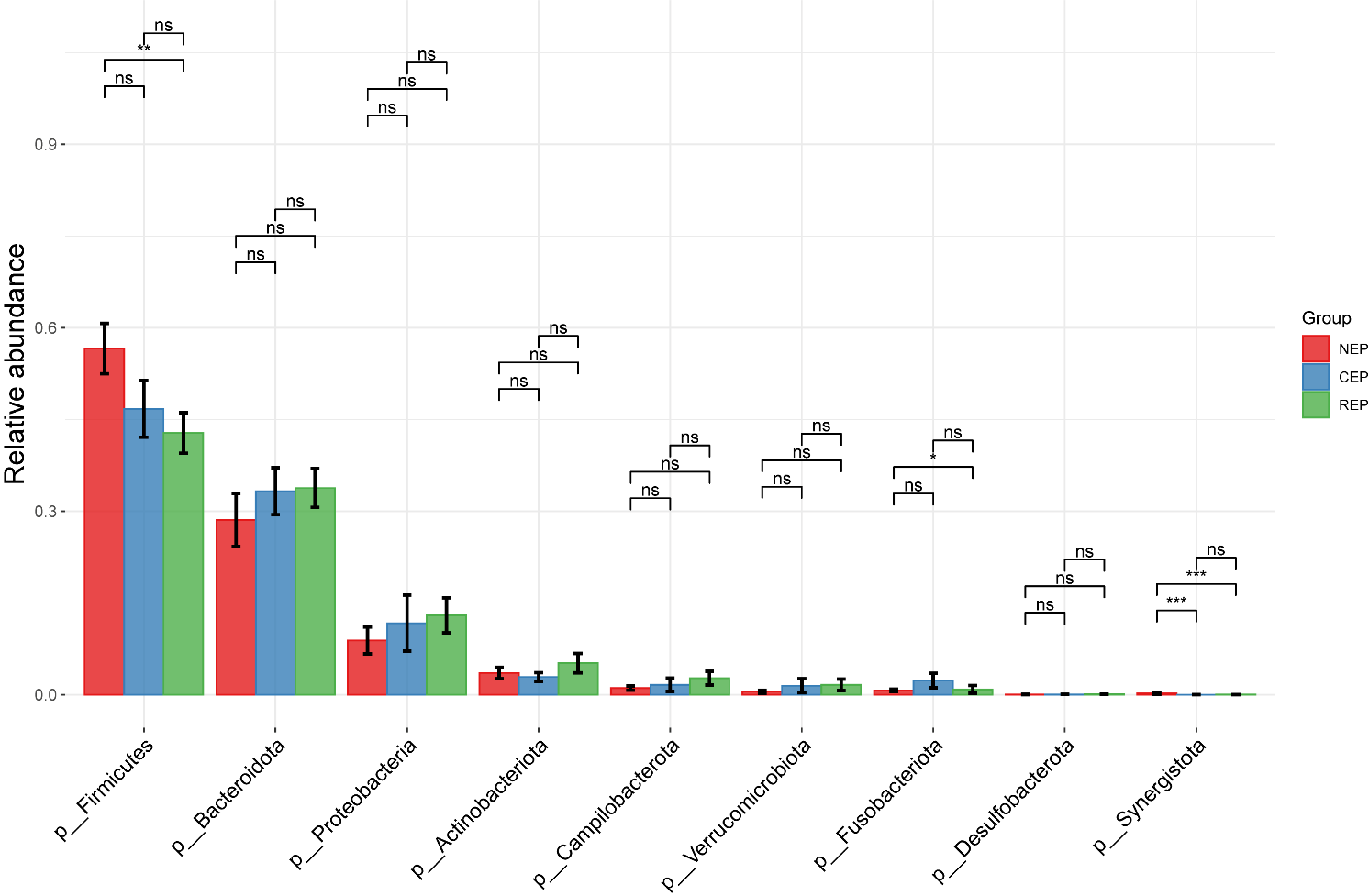


**Supplementary Figure S1. Differences analyzed at phylum level of microbiota (the top nine phyla) among three groups.** The p-values were calculated using a Wilcoxon rank sum test as implemented in the microeco package to evaluate the significance of the microbiota at phylum level between different groups. *, **, and *** indicate statistically significant differences at p < 0.05, p < 0.01, and p < 0.001, respectively.


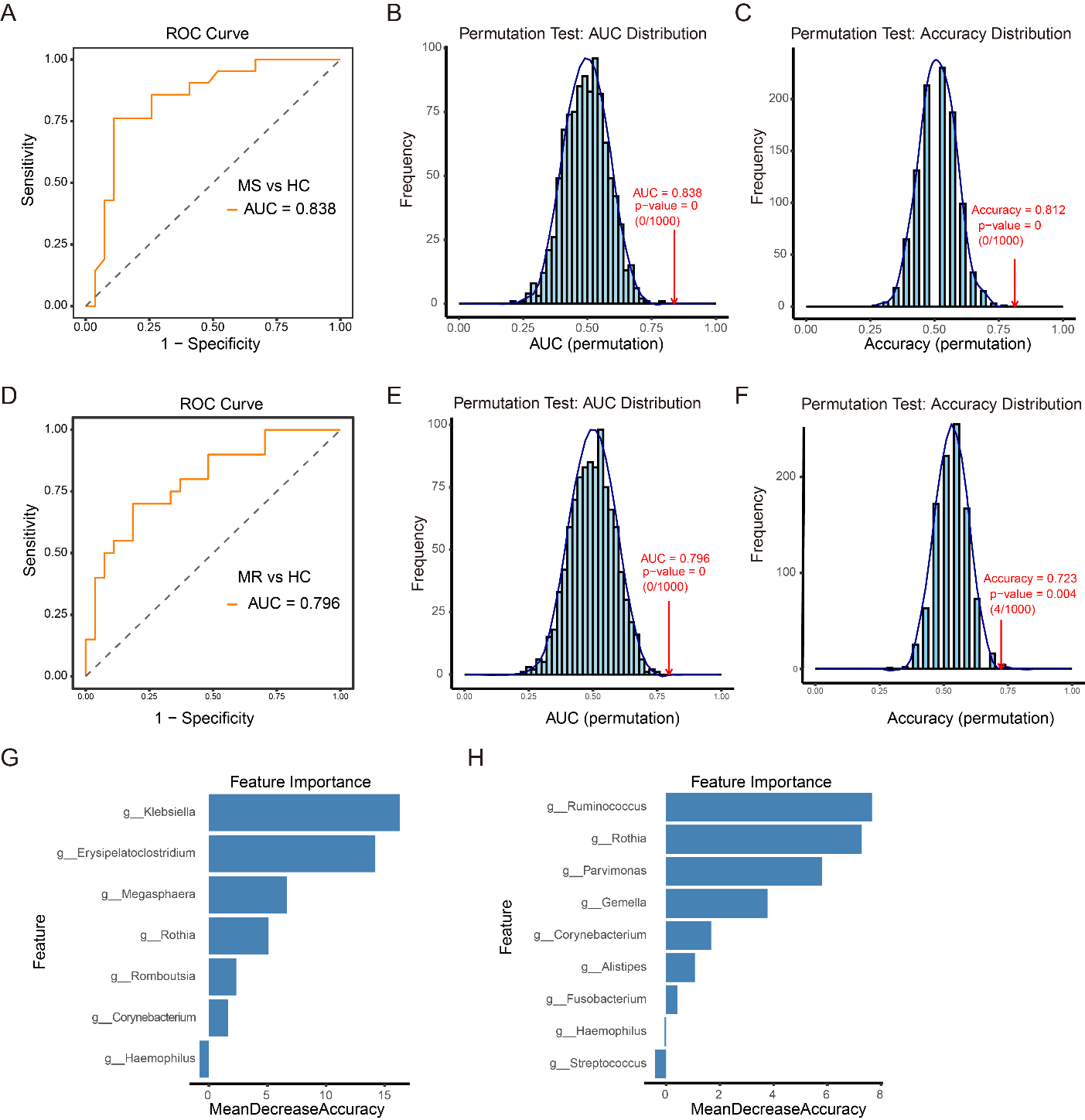


**Supplementary Figure S2. Cross-cohort validation of microbial classification models using an external pediatric epilepsy dataset from a European cohort.** Panels A–C present the performance of the CEP vs NEP classifier applied to medication-sensitive (MS) vs healthy control (HC) samples: ROC curve (A), permutation test results for AUC distribution (B), and permutation test results for accuracy distribution (C). Panels D–F show corresponding results for the REP vs NEP classifier applied to medication-resistant (MR) vs HC samples: ROC curve (D), AUC permutation test (E), and accuracy permutation test (F). Panel G: feature importance for CEP model with retained taxa highlighted; Panel H: feature importance for REP model.


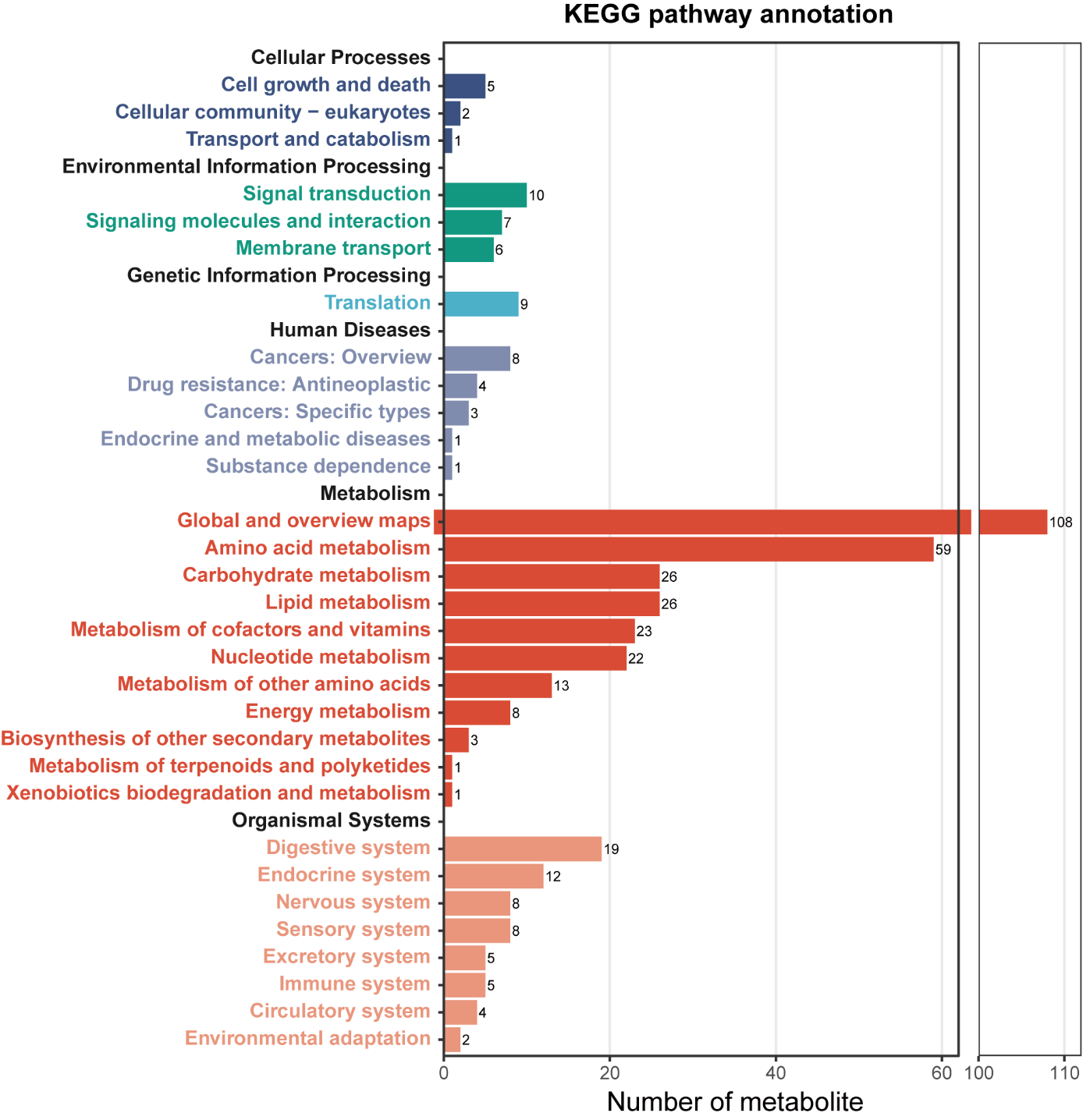


**Supplementary Figure S3. Functional classification of identified metabolites based on KEGG pathway annotation.** Bar chart depicting the distribution of annotated metabolites across major KEGG functional categories. The number of metabolites linked to each category is indicated, highlighting their relative representation within the dataset.


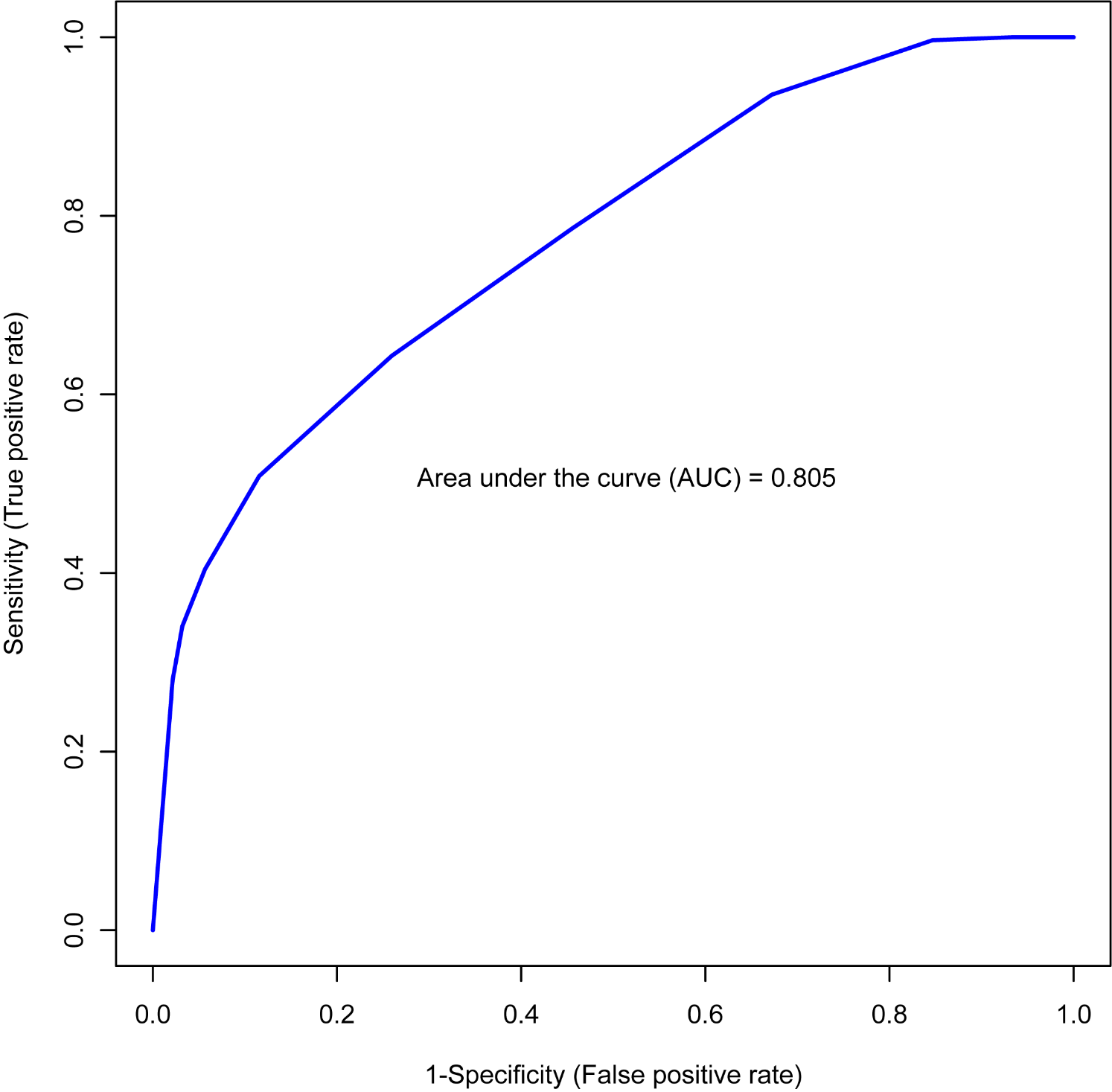


**Supplementary Figure S4. The ROC curve of the PLS-DA model based upon its average cross validation performance for all metabolites.** The ROC area was 0.848 (95% CI: 0.589–0.986).
